# Does gambling preference level affect occupational fraud behavior?—Evidence from a survey study in China

**DOI:** 10.3389/fpsyg.2025.1494990

**Published:** 2025-02-06

**Authors:** Shihua Huang, Yizao Chen, Baitong Li

**Affiliations:** ^1^School of Economics and Management, Shenyang Agricultural University, Shenyang, China; ^2^School of Accounting, Shandong University of Finance and Economics, Jinan, China; ^3^School of Management, Tianjin University of Technology, Tianjin, China

**Keywords:** gambling preference, occupational fraud, fraud triangle theory, pressure, ego depletion

## Abstract

Occupational fraud presents significant economic challenges globally. This study aims to understand the factors contributing to such fraudulent behavior and to develop strategies to mitigate it, focusing on the relationship between gambling preferences and occupational fraud within the framework of the fraud triangle theory, emphasizing the ‘pressure' element. To explore this relationship, the research employed several methods, including reliability and validity tests, correlation analysis, and regression analysis, to strengthen the survey research. The findings indicate that individuals with stronger gambling preferences are more likely to engage in occupational fraud. This relationship is mediated by ego depletion and moderated by psychological capital and superstitious beliefs, which align with theoretical models of cognitive biases. Further analysis reveals that psychological capital and superstitious beliefs play a moderating role through the mediating effect pathway from gambling preferences to ego depletion. The study provides valuable insights for developing effective fraud prevention strategies in corporate governance.

## 1 Introduction

Occupational fraud is a long-standing issue in the field of corporate governance, due to its high incidence and harmful and hidden characteristics. Undoubtedly, it represents a challenge the high-quality development of China's economy and hinders the construction process of modernization of national governance capacity and system. The “Country Reports on Functional Fraud and Abuse” (Association of Certified Fraud Examiners, 2022) reported that 2110 cases of functional fraud were investigated between January 2020 and September 2021, covering 133 countries and involving 23 industries. These cases caused economic losses of up to US$3.6 billion, with median and mean losses per case as high as US$117,000 and US$1.783 million, respectively. According to the results of the “China Corporate Employee Fraud Crime Judicial Adjudication Big Data Report,” 4,164 Chinese corporate employee fraud cases were found in 2020, an increase of 4.23% compared to the 3,995 cases in 2019. In 2020, 5,185 defendants were involved in fraud cases, an increase of 12.38% compared with 4,614 defendants in 2019. Overall, the total money involved in employee fraud cases in Chinese companies in 2020 was over 7.5 billion yuan, with an average of 1.8 million yuan. As China is currently in a critical stage of economic transformation, the economic consequences of occupational fraud could have a huge impact on the smooth operation of the national economy and the coordinated development of social wealth (Chen et al., 2016).

Given the harmfulness and prevalence of fraud, the basic question of “why do individuals commit fraud” has been discussed in depth by the theoretical and practical circles and has formed three famous theories of fraud motivation, respectively, the iceberg theory (Freud and Breuer, 1895), the fraud triangle theory (Cressey, 1953), and the fraud GONE theory (Bologna and Lindquist, 1993). Fraud Triangle Theory is the only theoretical tool that has been written into national auditing standards to guide the practice of fraud, and it is a classic and widely used theory of fraud motivation that defines three major conditions for fraud—pressure, opportunity, and rationalization. Our study focused on the pressure element of the fraud triangle. According to the ACFE survey (Albrecht et al., 1995), with the frequent occurrence of high-risk events worldwide, different social groups face different levels of pressure from maladaptive behaviors. While this study focuses on gambling preferences as a representative factor of pressure within the fraud triangle theory, it is important to acknowledge the role of risk decision-making as a behavioral trait. This study focuses on gambling preferences as a key aspect of stress-related behavior for the following reasons: (1) the rewards and costs of gambling are relatively straightforward to observe and measure; (2) the results of gambling (winning or losing) occur instantly; and (3) both winning and losing have certain probabilities. Thus, the level of gambling preference is more likely to reflect employees' trait preferences and behavioral characteristics. This study focuses on gambling preferences as a representative factor of pressure within the fraud triangle theory. The potential interplay between gambling preferences and risk decision-making, based on cognitive biases such as the illusion of control, may lead to unethical behaviors. The behaviors could ultimately result in occupational fraud. The potential influence of biases or irrational beliefs specific to gambling on occupational or work environments may be explained by certain pathways. For instance, gambling-related cognitive biases, such as illusions of control, might subtly affect professional decision-making by leading individuals to overestimate their ability to control outcomes in work scenarios. Additionally, habitual risk-seeking behaviors cultivated in gambling contexts could shape tendencies toward unwarranted risk-taking under pressure. Importantly, ego depletion, caused by gambling-related stress and the depletion of self-control resources, could act as a key mediator, reducing individuals' ability to resist unethical temptations in professional settings. These tendencies, influenced by gambling preferences, may increase the likelihood of behaviors that culminate in occupational fraud.

The central focus of this study is to examine, based on survey data from China, whether corporate actors are predisposed to engage in occupational fraud due to their personal gambling preferences. It is important to clarify that the term “occupational fraud,” as used in this research, primarily refers to inappropriate actions undertaken by employees within an organization driven by individual traits or external pressures, such as embezzlement, falsification of expense claims, or misappropriation of company resources. This definition is distinct from organizational-level fraud, such as financial statement manipulation by executives or collusive fraud. The study specifically emphasizes individual-level occupational fraud, particularly labor-related misconduct.

Based on the above analysis, this study adopts gambling preference as a representative factor of pressure, based on the pressure element within the fraud triangle theory, and examines its mechanism of influence on occupational fraud decision-making through ego depletion. Specifically, the depletion of self-control resources may intensify irrational gambling beliefs from the perspective of cognitive bias, thereby leading corporate actors to engage in high-risk behaviors when confronted with financial pressures, to satisfy gambling needs or to compensate for economic shortfalls. Thus, this study attempted to reveal the source of the influence of gambling preference level on occupational fraud behavior by systematically selecting and constructing appropriate measurement tools and adopting a survey research method to empirically examine the mechanism of the role of gambling preference on the occupational fraud behavior of corporate actors. We aimed to be able to propose inhibition strategies that can be used as paths of action and mechanisms of influence. This study makes several contributions. First, the existing literature on fraud is dominated by theoretical studies and empirical studies based on econometric methods, and the current study provides theoretical and practical scenarios for the identification and measurement of occupational fraud pressure, realizing the cross-fertilization of psychology, economics, and management. Second, based on the “limited rationality” hypothesis, the study can clarify the important position of corporate employees' gambling preference and irrational beliefs in the decision-making related to fraudulent behaviors. It can also provide new ideas for preventing fraudulent behaviors from the perspective of individuals traits to make up for the governance deficiencies of the traditional external regulatory means and help improve the strategic decision-making program in line with the scenarios of fraudulent behaviors.

## 2 Literature review

### 2.1 Factors influencing occupational fraud

Research has focused on identifying the factors that influence the prevalence of occupational fraud at the societal, corporate, and executive levels.

#### 2.1.1 Factors influencing occupational fraud behavior at the societal level

Occupational fraudulent behavior is affected by the social environment, and existing studies have analyzed this behavior from three perspectives—the institutional environment, industry characteristics, and regional environment. In terms of institutional pressure, Oliver (1991) found that the delisting system prompted listed companies to have a strong motive to manipulate profits to achieve profitability effects, breeding financial reporting fraud. The party organization-embedded system (Chen and Zhang, 2022), inspection system (Zhang and Zhang, 2020), and random inspection system (Ban et al., 2022) can play a regulatory role and suppress corporate violations, corruption, and other fraudulent behaviors. Second, from the industry environment level, the stronger the degree of product competition, the higher the chance that listed companies will have fraud or violation motives (Teng et al., 2016). Ndofor et al. (2015) found that information asymmetry caused by industry complexity increased financial reporting fraud. Finally, at the regional level, research has demonstrated that the better the degree of marketization or economic level (Zhang and Ma, 2005), the higher the credit level (Dong et al., 2018), and the lower the probability of corporate occupational fraud. Conversely, Wang (2021) examined the relationship between lottery culture and corporate fraud from the perspective of local culture and found that the stronger the lottery culture of a region, the higher the tendency toward adventurism and risk tolerance and the higher the probability of corporate financial fraud.

#### 2.1.2 Factors influencing occupational fraud behavior at the corporate level

Financial difficulties faced by enterprises are a key driver of fraudulent behavior. At the enterprise level, fraudulent pressures include the whitewashing of financial statements driven by performance evaluations and target pressures (Ge, 2003). He et al. (2015) found that corporate executives are incentivized by upward comparisons, making some companies strive to be the first when formulating strategic planning, and this kind of catch-up-oriented business strategy can bring pressure to catch up with the company, potentially impacting company's violations, fraud, and other negative behaviors. In addition, Peng et al. (2021) also found that following the stressor-emotion theory, when a company lacks an adequate reward mechanism, employees are more likely to be emotionally exhausted, which will directly and positively affect the occurrence of employees' unethical behavior.

#### 2.1.3 Factors influencing occupational fraud at the executive level

Studies have examined the effects of the executive power structure (Chen et al., 2017), executive gender structure (Gan et al., 2015), executive background characteristics (Lu et al., 2015), and a lack of board function (Su and Yin, 2010) on the governance of firms' occupational fraud. Li et al. (2021) found that firms with risk-averse CEOs are more likely to violate rules. Hu et al. (2022) reported that the degree of management overconfidence is positively related to the occurrence of corporate violations.

### 2.2 Studies related to gambling

Gambling is a multifaceted disorder influenced by a range of factors, with cognitive biases or distortions identified as a key contributor to the development of problematic gambling. These cognitive distortions are considered significant drivers of gambling behavior, as they impair decision-making in situations involving risk or uncertainty (Cocker and Winstanley, 2015). Representative data from the United States, Canada, and the United Kingdom indicate that 72%−86% of the population engages in gambling to varying degrees (Gerstein et al., 1999; Wardle et al., 2012; Azmier, 2000). The accessibility of gambling has further increased with the proliferation of internet gambling, fantasy sports, and large-scale lotteries such as Powerball, leading to its growing prevalence (Griffiths, 1999). While the motivations for gambling are diverse, the pursuit of financial gain remains a significant factor (Neighbors et al., 2002). However, cognitive theories of gambling suggest that the progression from recreational gambling to problematic gambling, and the escalating preference for gambling, are primarily driven by an increase in irrational or distorted beliefs about the outcomes of uncertain events (Walker, 1992).

Employees with a strong inclination toward gambling may experience a decline in work efficiency, which can, in certain situations, escalate into financial pressures. These pressures may manifest as borrowing money from colleagues and, in some cases, even extend to theft of workplace property or embezzlement (Binde, 2016). Research on pathological gamblers reveals that their gambling-related issues often present distinct workplace risks: 74% of work-related problems were directly linked to gambling, including inattention (57%), high rates of sick leave (46%), and frequent borrowing of money at work (11%). Moreover, a smaller proportion of cases involved workplace theft, fraud, or misappropriation of funds (14%). The study further found that 12% of individuals faced dismissal due to these issues, while 29% voluntarily resigned (Bergh and Kühlhorn, 1994). A key characteristic of both social and pathological gambling is loss aversion, which manifests as “chasing losses”—a behavior where individuals persist in gambling to recover lost funds (Lesieur, 1979). Furthermore, gambling behaviors are closely associated with elevated risk-taking tendencies, which may independently drive occupational fraud beyond cognitive biases such as the illusion of control (Mishra et al., 2010). Research by Binde (2016) highlights that gambling-related cognitive distortions, combined with risk-seeking behavior, can exacerbate unethical tendencies. This connection suggests that gambling preferences could indirectly reflect broader risk decision-making tendencies, highlighting the need for more nuanced research on how these factors interact to influence occupational fraud.

### 2.3 Literature evaluation

The current research on occupational fraud from a motivational perspective has identified three levels of factors affecting the likelihood of occupational fraud—society, enterprise, and executives. However, the existing studies have some shortcomings. First, few studies have considered individual cognitive factors, and existing studies have not examined the impact of individual decision-making on fraudulent behavior by designing appropriate fraud scenarios. Second, although some scholars have attempted to conduct research on actors' fraudulent decision-making using econometric methods, they can only judge and identify the risk of fraud at the level of capital data indicators and cannot specifically and systematically analyze the causes of fraudulent behavior in the workplace. Furthermore, most studies focusing on the level of fraud motivation/pressure remain conceptual and theoretical, and measures for the relevant variables have not yet been standardized. Even though the literature recognizes that gambling breeds risky behaviors, it has not focused on the level of fraudulent behaviors in the workplace. Given existing research, this study used a questionnaire survey that was analyzed using regression and other research methods to explore the influence of the gambling preference of pressure on occupational fraud behavior. The study aims to identify the stress factors influencing individuals' decision-making processes, provide systematic empirical evidence for the practical applicability of the classical fraud triangle theory at a specific dimensional level, and contribute to the development and refinement of fraud governance strategies.

## 3 Research hypotheses

### 3.1 Gambling preferences and occupational fraud

The development and evolution of gambling preferences are often associated with illusions of control and irrational cognition (Armstrong et al., 2020). Illusions of control refer to an individual's tendency to overestimate their influence on the outcome of events, leading them to subjectively believe that the probability of success is higher than the objective reality (Thompson et al., 1998). This phenomenon is more likely to occurs when an individual has a strong desire for a particular outcome or perceives a direct connection between their actions and the outcome (Langer, 1975). Such illusions are often sustained through biased attribution processes, such as a tendency to attribute success to one's abilities while attributing failure to external factors (Gilovich and Douglas, 1986). In gambling activities, this cognitive bias might contribute to inflated expectations of winning, thereby perpetuating or even intensifying gambling behavior (Baboushkin et al., 2001). Sharpe (2002) suggested that these irrational cognitions could be activated by gambling-related cues and reinforced through learning, significantly driving new gambling behaviors or extending the duration of existing ones. McCusker and Gettings (1997) proposed that these cognitive biases operate automatically and may trap individuals in a cycle of losing control over their gambling behavior.

In workplace settings, individuals with strong gambling preferences may exhibit a tendency to overestimate their abilities and control over the outcomes of their actions (Bouju et al., 2014). Consequently, such illusions of control might lead them to underestimate the risks or consequences of failure when presented with opportunities for fraud. For instance, in financial operations, these individuals may believe they can manipulate processes or bypass rules to gain benefits without detection. This overconfidence has the potential to increase the likelihood of engaging in behaviors such as embezzlement or false reimbursement claims, driven by the desire to achieve higher returns. From a theoretical perspective, the connection between gambling preferences and occupational fraud can be understood through the progressive influence of cognitive biases. Gambling-related illusions of control may shape an individual's overall attitude toward risk and decision-making, increasing their willingness to engage in unethical behavior, especially when the perceived benefits outweigh the risks. This process aligns with the theory of cognitive biases, suggesting that individuals are more likely to act on distorted perceptions when under pressure or motivated by personal gain. Based on the above analysis, the following hypothesis is proposed:

H1: Individuals with stronger gambling preferences are more likely to exhibit behaviors associated with occupational fraud.

### 3.2 The mediating role of ego depletion

Ego depletion refers to a state in which an individual's self-regulatory resources are reduced, potentially leading to insufficient capacity or willingness for self-control (Hagger et al., 2010). Since self-regulatory resources are inherently limited, maintaining effective self-control in the face of external stimuli requires resource consumption, which could eventually results in ego depletion (Baumeister et al., 1998). Individuals with a gambling preference might be driven by irrational beliefs such as the “illusion of control,” which require frequent emotional regulation and repeated attempts to recover losses. This process may deplete self-regulatory resources over time (Lakey et al., 2008; Parikh, 2018; Raylu and Oei, 2004). When in a state of ego depletion, individuals experience may have diminished self-control and moral judgment, as the depletion of resources weakens their ability to regulate internal urges. In this state, they might be more inclined to engage in hasty actions (Muraven and Baumeister, 2000) and be influenced by risky decision-making (Baumeister, 2003). Ego depletion could also impairs individuals' resistance to external temptations in the workplace, potentially making them more inclined to pursue immediate rewards, such as borrowing money, stealing resources, or engaging in financial misconduct (Binde, 2016). Additionally, ego depletion may reduce an individual's capacity to assess risks (Kouchaki and Smith, 2014), possibly causing them to prioritize short-term gains over long-term consequences (Gino et al., 2011), thereby increasing the likelihood of fraudulent behavior. Based on this analysis, this study posits that ego depletion may acts as a mediating factor between gambling preference and occupational fraud. Individuals with a high gambling preference might be more prone to experiencing ego depletion due to the psychological and emotional demands associated with gambling. This state could weaken their ability to resist fraudulent behaviors, potentially increasing the risk of occupational fraud. Based on the above analysis, the following hypothesis is proposed:

H2: Ego depletion would mediate the relationship between gambling preference and occupational fraud.

### 3.3 The moderating role of psychological capital

Psychological capital is a positive mindset consisting of four dimensions—self-efficacy, hope, optimism, and resilience (Luthans and Youssef, 2004)—and is considered a protective factor for social adaptation (e.g., an employee with high psychological capital might stay motivated and perform effectively even after a failed project, demonstrating resilience and adaptability in challenging situations). As a positive energy resource, as a positive psychological resource, psychological capital may help mitigate the impact of negative behaviors. Psychological capital has been found to protect individuals' positive emotions, and research has identified an association between happiness and satisfaction in people's lives (Wang and Wang, 2013) and alleviate certain negative psychological outcomes (Sun et al., 2013). These findings suggest that psychological capital might serve as a resource that moderates the relationship between individuals and undesirable behaviors. In the context of this study, psychological capital may reduce the strength of the association between gambling preferences and occupational fraud. Based on these theoretical considerations, we hypothesized:

H3: Psychological capital would moderate the relationship between gambling preference and fraudulent occupational behavior. The stronger the psychological capital, the weaker the positive relationship between gambling preference and the tendency toward occupational fraud.

### 3.4 The moderating role of superstitious beliefs

Superstition is fundamentally a psychological phenomenon, and its underlying mechanism may be explained through irrational beliefs rooted in cognitive biases (Teovanović et al., 2024; McInnes et al., 2014). Gambling behavior, characterized by its reliance on luck, inherently involves significant uncertainty. This uncertainty could heighten anxiety levels, particularly among individuals with a stronger preference for gambling (Mueller et al., 2010; Miu et al., 2008). Under stress or adversity, individuals might adopt irrational coping strategies, including superstitious beliefs, to create a perceived “sense of control” over their circumstances. This reliance on superstitions could contribute to deviant behaviors (Torgler, 2007). Superstitious beliefs may bolster an individual's confidence in “luck” or “supernatural forces,” potentially causing them to disregard the negative consequences associated with gambling (Joukhador et al., 2004). For individuals with a high gambling preference, these beliefs may amplify risk-taking tendencies, which could increase the likelihood of engaging in unethical actions—such as embezzling company funds—to satisfy gambling needs or recover losses. These irrational beliefs might offer a cognitive rationalization for occupational fraud, leading individuals to potentially disregard the moral implications of their actions (Gino et al., 2011). In this process, superstitious beliefs strengthen the influence of gambling preferences, increasing the likelihood of engaging in fraudulent behavior. Based on the above analysis, the following hypothesis is proposed:

H4: Superstitious beliefs would moderate the relationship between gambling preference and occupational fraud behavior. The stronger the superstitious beliefs, the stronger the positive association between gambling preference and the tendency toward occupational fraud.

**Figure 1:**
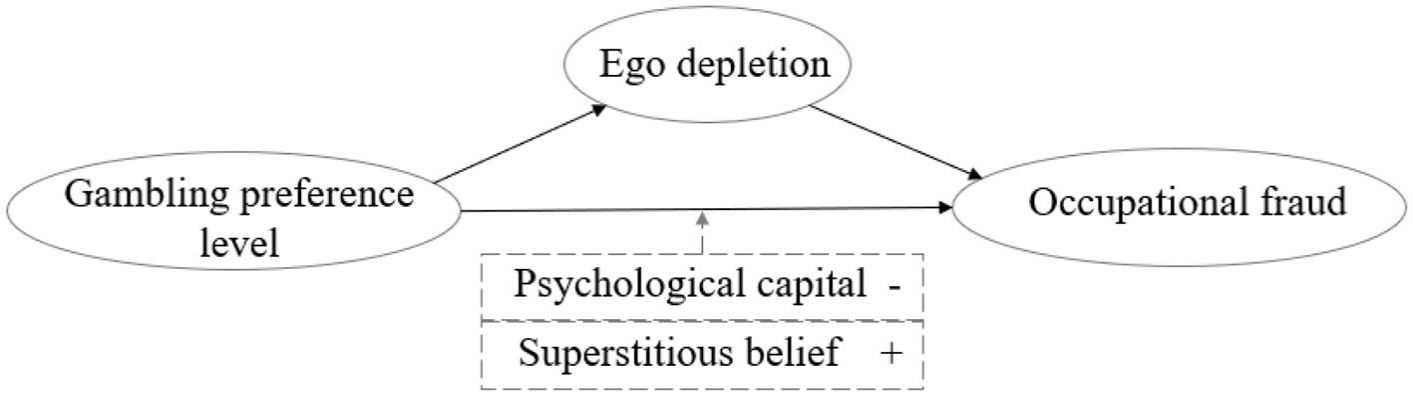
The impact and mechanism of action pathway of gambling preference level on occupational fraud behavior.

The specific hypothesized pathways are shown in Figure 1.

## 4 Research design

### 4.1 Study sample and data sources

The study's target population was enterprise staff, which was mainly distributed online through the Chinese Survey System, Questionnaire Star. The questionnaire was answered anonymously, and participants were told that the information obtained from this questionnaire is only used for academic research, there were no right or wrong answers, and everything was confidential. Ultimately, 679 questionnaires were collected. This paper excludes 12 questionnaire responses completed in less than 40 s and 132 responses that failed the quality test^1^ to ensure the validity of the questionnaires. The validity of the questionnaires used for the research test is 535, and the validity rate of the questionnaire is 78.79%. The basic sociodemographic characteristics of the sample showed that 46.17% of the sample was male and 53.83% was female. Those aged 18–25 years was 7.10%, those 26–30 years was 35.14%, aged 31–40 years was 40.37%, aged 41–50 years was 8.41%, 5.41% were aged 51–60, and 3.74% were aged 60 and older. In terms of education, the proportion of the sample with high school education and less was 4.30%; those who were a specialist were 9.53%; those with bachelor's degrees were 27.48%, and those who had postgraduate degrees represented 58.69% of the sample. For job rank, the proportion of the sample who were ordinary employees as 74.21%, the proportion who were managers was 19.81%, and the proportion who were senior managers was 5.98%. In terms of the nature of ownership of the enterprises in which they worked, 30.65% were working at state-owned enterprises, and 69.35% were at non-state-owned enterprises.

### 4.2 Definition of variables

#### 4.2.1 Explained variables

Owing to the sensitivity of measuring of occupational fraud and the inability to obtain the desired results through direct questioning, a scenario simulation was implemented. Cavanagh and Fritzsche (1985) found that scenario simulation was more valid than other research tools for the measurement of ethical variables and that scenario simulation provided more realistic decision-making situations and elicited more realistic action responses from behavioral decision-makers, making it more valid than other research tools. In addition, scenario simulation can effectively measure the internal micro changes of the participants in the process of making fraudulent decisions, which is reflected in the indicator of the “propensity to engage in fraudulent behavior.” Ajzen's (1991) study defines the propensity to engage in fraudulent behavior in the workplace as a type of reactive intention in the field of behavioral decision-making research and a direct prerequisite for the occurrence of fraudulent behavior. The higher the propensity to commit fraud, the higher the actual incidence of fraudulent behavior by corporate actors. According to the existing research, most measurements of occupational fraud come from capital market data that examined fraud or violation incidents that have occurred or been disclosed, the number of recorded occupational frauds, or the results of the implementation or non-implementation of occupational fraud by enterprises or executives through the dichotomy method. The above measurement models cannot predict fraudulent decisions before they occur, nor can they observe the psychological decision-making process before the perpetrator commits a fraudulent act. Therefore, to understand in detail the internal changes of the subjects in the decision-making process of fraud, this study adopts the scenario simulation method and other methods to portray the specific research variables.

##### 4.2.1.1 Fraud scenario scale

To accurately measure the tendency of corporate actors to engage in fraudulent behavior in specific scenarios, we referred to Ng et al. (2009) and other fraud scenarios to consider the context of China's reality to design a fraud scenario scale. The main items included, “whether an employee has a hidden tendency to exploit their position for personal gain; whether an individual facing urgent financing needs but failing to meet bank credit requirements attempts to use special methods to qualify; and whether an individual attempts to inflate accounts to achieve personal objectives.” Through pre-research, structural validation, and refinement based on similar studies, three scenarios were designed, and items were rated using a 7-point Likert scale. This scale utilized Cronbach's alpha coefficient to assess the internal consistency of the questionnaire data for reliability analysis. For validity analysis, the KMO statistic was employed to evaluate whether the questionnaire data had a robust factor structure. The scale has a Cronbach's alpha coefficient of 0.704 and a KMO value of 0.662, which is significant at the 1% level, indicating that it passed the reliability and validity tests.

##### 4.2.1.2 Questionnaire quality control

The research process was based on the Fraud Scenario Scale, with the addition of a post-situational check to determine how well participants understood the rules for completing the scale, how attentive they were when answering the questions, and to retain the current sample when the questions were answered correctly.

#### 4.2.2 Explanatory variables

##### 4.2.2.1 Gambling irrational beliefs scale

The Chinese version of the (GRCS-C) was developed Raylu and Oei (2004) and consists of the following factors: control illusion (e.g., “Praying increases my chances of winning”), predictive control (e.g., “When I lose a bet, I'm sure to win next”), gambling expectancy (e.g., “I want to continue to win after winning”), interpretive bias (e.g., “My loss is only related to probability factors such as bad luck”). In this study, based on the scale's factors, we introduced the items related to welfare lottery, scratch-off lottery, sports betting, and Mahjong poker, which are suitable for gambling scenarios in China, and redesigned the related measures of gambling preference. These items included statements such as, “Before scratching off a lottery ticket, I pray and hope to win a big prize,” “when I lose some money playing poker with friends, I want to play again to win it back,” and “when I win a small prize in a lottery, I feel like continuing to buy tickets, hoping to win more money” from among the five items. These items were scored on a 7-point Likert scale. This scale has a Cronbach's alpha coefficient of 0.842 and a KMO value of 0.851, which is significant at the 1% level, indicating that it passed the reliability and validity tests.

#### 4.2.3 Mediating variables

##### 4.2.3.1 Ego depletion scale

Drawing on the state-based self-control scale designed by Lindner et al. (2015). This scale was designed to represent the degree of ego depletion by measuring an individual's self-control. The higher the value, the stronger the state of ego depletion of the corporate actor. To ensure the questionnaire remained concise and focused while retaining its validity, we streamlined the scale to five items, such as, “It is difficult for me to resist all kinds of temptations now,” “if I am given a difficult task now, I will not be able to do it,” “I feel lazy now,” “it is difficult for me to digest any information now,” and “I feel that my willpower is very weak now.” These questions were scored on a 7-point Likert scale. The scale has a Cronbach's alpha coefficient of 0.884 and a KMO value of 0.860, which is significant at the 1% level, indicating that it passed the reliability and validity tests.

#### 4.2.4 Moderating variables

##### 4.2.4.1 Psychological capital scale

The Psychological Capital Scale is based on the Psychological Capital Scale developed by Ke et al. (2009). We limited the length of the questionnaire that we used to five items, namely, Transactional Psychological Capital (4 items) and Interpersonal Psychological Capital (1 item), such as “I like to set higher goals for myself,” “I believe that I am capable of doing my job” “I will laugh away when others say something unfavorable to me,” “I always think in a good direction when I am not sure of the outcome,” and “I always think in the right direction.” The questions were scored on a Likert 7-point scale, with higher scores indicating higher psychological capital. The scale has a Cronbach's alpha coefficient of 0.884 and a KMO value of 0.860, which is significant at the 1% level, indicating that it passed the reliability and validity tests.

##### 4.2.4.2 Superstitious belief scale

The Paranormal Belief Scale (PBS) by Tobacyk and Milford (1983). In this study, the scale was revised based on the work of Li et al. (2006). We selected five items from the traditional secular superstition dimension of the scale that were likely to have the greatest effect on the gambling dimension. The items included, “The number 4 is unfortunate,” “one can predict one's future marriage and career through horoscopes,” “wearing red in the year of one's birth can protect one from evil spirits and disasters,” “it is not suitable for a couple to get married if they are not compatible with each other's zodiac sign,” and “going to the temple to ask for a sign or make a wish at certain times is necessary.” The questions were scored on a 7-point Likert scale. The scale has a Cronbach's alpha coefficient of 0.866 and a KMO value of 0.862, which is significant at the 1% level, indicating that it passed the reliability and validity tests.

#### 4.2.5 Control variables

Control variables may influence the explained variables along with the main variables in this study. Therefore, in this study, basic sociodemographic information such as sex, age, education, years of working experience, industry, nature of the organization, job rank, and annual income of the respondent were selected as the control variables to be measured. In addition, the possible influence of opportunity and rationalization excitement factors on the findings of this study were controlled according to the fraud triangle theory, in which the probability of being detected was set at 15% when designing the occupational fraud scenario. According to the analytical design of Chen et al. (2019), five viewpoint items were selected for the rationalization excuse scale (e.g., “fabricating information might be a business strategy,” “if fraud is committed due to pressure from superiors, it can be excused,” and “it's understandable to accept bribes if it's the norm in the company culture”). The questions were scored on a 7-point Likert scale. The scale has a Cronbach's alpha coefficient of 0.807 and a KMO value of 0.808, which is significant at the 1% level, indicating that it passed the reliability and validity tests. The definitions of the study variables are listed in Table 1.

**Table 1:** Definition of variables and definitions.

**Variable**	**Notation**	**Definition**	
Explained variables	Tendency toward occupational fraud	*Fraud*	Factorized synthetic scores for the propensity to commit fraud in office items
Explanatory variables	Gambling preference	*Gamb*	Factorized synthetic scores for the gambling irrational beliefs question items
Mediating variables	Ego depletion	*Ego*	Factorized synthetic scores for ego depletion question items
Moderating variables	Psychological capital	*Psycap*	Factor synthesis scores for psychological capital question items
	Superstitious belief	*Belief*	Factorized synthetic scores for superstitious belief question items
Control variables	Gender	*Gender*	0 for women and 1 for men
	Age	*Age*	Below 18, 18–25, 26–30, 31–40, 41–50, 51–60, above 60 in order of value 1–7
	Educational level	*Educa*	High school and below, college, undergraduate, graduate students in order of value 1–4
	Years of work experience	*Works*	Less than 1 year, 1–3, 4–6, 7–10, 11–15, 16–20, more than 20 years in order of value 1–7
	Position level	*Rank*	Ordinary employees, managers, and top executives in order of value 1–3
	Income level	*Rev*	Annual income less than 50,000, 5–7, 8–10, 11–15, 16–20, 21–30, and more than 300,000 in order of value 1–7
	Ownership type	*Equity*	0 for non-state-owned enterprises, 1 for state-owned enterprises
	Rationalization	*Rat*	Factorized synthetic scores for rationalization excuse question items
	Industry	*Industry*	29 dummy variables based on Questionnaire Star's default industry classification and data structure

Factor analysis was used to synthesize the metrics for each subscale. The matrix of component score coefficients was calculated to determine the factor weights, and the synthesized expressions are shown in Equations 1–6. *Fraud* stands for occupational fraud tendency, *Gamb* stands for gambling preference, *Ego* stands for ego depletion, *Psycap* stands for psychological capital, *Belief* stands for superstitious beliefs, *Rat* stands for rationalization excuses, and *Fraud*_*n*_, *Gamb*_*n*_, *Ego*_*n*_, *Psycap*_*n*_, *Belief*_*n*_, and *Rat*_*n*_ stand for the *nth* question item of the corresponding factor.


(1) $$\begin{array}{l} Fraud\;=\;0.398Fraud_{1}\;+0.453Fraud_{2}\;+0.44\;Fraud_{3} \end{array}$$

(2) $$\begin{array}{l} Gamb=\;0.174Gamb_{1}\;+0.274Gamb_{2}\;+\;0.281Gamb_{3}\\ +\;0.267Gamb_{4}\;+\;0.250Gamb_{5} \end{array}$$

(3) $$\begin{array}{l} Ego=\;0.216Ego_{1}\;+0.250Ego_{2}\;+0.226Ego_{3}\\ +0.257Ego_{4}\;+0.253Ego_{5} \end{array}$$

(4) $$\begin{array}{l} Psycap=\;0.242Psycap_{1}\;+0.255Psycap_{2}\;+0.261Psycap_{3}\\ +0.246Psycap_{4}\;+0.245Psycap_{5} \end{array}$$

(5) $$\begin{array}{l} Belief=\;0.235\;Belief_{1}\;+0.252\;Belief_{2}\;+0.252\;Belief_{3}\\ +0.251\;Belief_{4}\;+0.248\;Belief_{5} \end{array}$$

(6) $$\begin{array}{l} Rat\;=\;0.236\;Rat_{1}\;+\;0.194\;Rat_{2}\;+\;0.288\;Rat_{3}\;+\;0.296\;Rat_{4}\\ +\;0.286\;Rat_{5} \end{array}$$

### 4.3 Model specifications

We constructed equations to test our hypotheses (H1–H4). Equation (7) was used to test hypothesis H1 (i.e., the relationship between the gambling preference level and occupational fraud). Equations (8) and (9) were used to test hypothesis H2 (i.e., the mediating role of ego depletion between the gambling preference and occupational fraud. Equation (10) was used to test hypothesis H3 (i.e., the moderating role of psychological capital in gambling preference and occupational fraud behavior). Equation (11) was used to test hypothesis H4 (i.e., the moderating role of superstitious beliefs between gambling preference level and occupational fraud behavior).


(7) $$\begin{array}{l} Fraud_{i}=\alpha_{0}+\alpha_{1}Gamb_{i}+\sum \alpha Control_{i}+\varepsilon_{i} \end{array}$$

(8) $$\begin{array}{l} Ego_{i}=\beta_{0}+\beta_{1}Gamb_{i}+\sum \beta Control_{i}+\varepsilon_{i} \end{array}$$

(9) $$\begin{array}{l} Fraud_{i}=\gamma_{0}+\gamma_{1}Gamb_{i}+\gamma_{2}Ego_{i}+\sum \gamma Control_{i}+\varepsilon_{i} \end{array}$$

(10) $$\begin{array}{l} Fraud_{i}=\varphi_{0}+\varphi_{1}Gamb_{i}+\varphi_{2}Psycap_{i}+\varphi_{3}{Gamb_{i}\times Psycap}_{i}\\ +\sum \phi Control_{i}+\;\varepsilon_{i} \end{array}$$

(11) $$\begin{array}{l} Fraud_{i}=\omega_{0}+\omega_{1}Gamb_{i}+\omega_{2}Belief_{i}+\omega_{3}{Gamb_{i}\times Belief}_{i}\\ +\sum \omega Control_{i}+\varepsilon_{i} \end{array}$$

In the above model, *i* refers to the different study subjects, and *Control* refers to the control variables.

### 4.4 Common method variance

The research method of Zhou and Long (2004) was used to analyze the common method variance of the scale data in this study, primarily including two kinds of control for the procedure and the statistical level. Among them, procedural control mainly includes ensuring that the participants are anonymous and having control over the time of acquiring the sample data. In the statistical control, Harman's one-way test was adopted, which is usually accomplished by using the exploratory factor analysis (EFA) method, referring to the degree of variation in the different items that can be explained by a single factor. The higher the degree of variation that can be explained by a single factor, the more serious the problem of homologous bias is. According to Podsakoff and Organ (1986), if a single factor identified by exploratory factor analysis (non-rotated) does not explain more than 50% of the variance, then the homoscedasticity bias problem is not significant. Our findings showed that the total explanatory power of the common factor variables was 57.760%, and the explanatory power of the non-rotated first factor was 24.626%, which is lower than the estimation criterion of 50%; therefore, there was no serious common method variance problem in this study.

## 5 Empirical results and analysis

### 5.1 Descriptive statistics

The descriptive statistics of the main variables are presented in Table 2. The mean value of gambling preference (*Gamb*) was 3.973 (range: 1.246–8.722), indicating that the questionnaire was well-designed and could measure the differences in gambling-related stress levels endured by different corporate actor samples. From the perspective of fraud decision-making, the mean value of the propensity to commit occupational fraud (*Fraud*) measured by scenario analysis is 4.093 (SD = 2.188), indicating that the participants had a high level of understanding of the situational variables of occupational fraud and could measure real fraud intention according to their own situation. The mean value of ego depletion (*Ego*) was 3.459 (range: 1.733–8.414), indicating that there are different degrees of ego depletion among participants, and this range reflects individual differences in the depletion of self-control resources, effectively capturing ego depletion in the sample. Among the moderating variables in this paper, the mean values of psychological capital (*Psycap*) and superstitious belief (*Belief* ) are 5.754 and 4.019, respectively. In terms of control variables, the mean value of gender (*Gender*) is 0.462, which indicates that the research sample is more balanced between men and women; and the mean value of rationalization excuse (*Rat*) is 4.072, with a minimum value of 1.300 and a maximum value of 9.100, indicating that the participants' acceptance of the rationalization excuse measure was high, and the distribution of the rest of the variables will not be redundantly repeated.

**Table 2:** Descriptive statistics of variables.

**Variable**	***N***	**Mean**	**SD**	**Min**	**p25**	**p50**	**p75**	**Max**
*Fraud*	535	4.093	2.188	1.291	2.171	3.931	5.507	9.037
*Gamb*	535	3.973	1.918	1.246	2.409	3.793	5.125	8.722
*Ego*	535	3.459	1.733	1.202	2.130	3.175	4.743	8.414
*Psycap*	535	5.754	1.868	1.249	4.979	5.996	7.01	8.743
*Belief*	535	4.019	1.990	1.238	2.242	4.197	5.246	8.666
*Gender*	535	0.462	0.499	0.000	0.000	0.000	1.000	1.000
*Age*	535	3.807	1.109	2.000	3.000	4.000	4.000	7.000
*Educa*	535	3.406	0.831	1.000	3.000	4.000	4.000	4.000
*Works*	535	3.942	1.721	1.000	3.000	4.000	5.000	7.000
*Rank*	535	1.318	0.581	1.000	1.000	1.000	2.000	3.000
*Rev*	535	3.867	1.826	1.000	2.000	4.000	5.000	7.000
*Equity*	535	0.307	0.461	0.000	0.000	0.000	1.000	1.000
*Rat*	535	4.072	1.969	1.300	2.464	3.948	5.200	9.100

To further describe the effect of gambling preference on occupational fraud behavior, we conducted independent samples *t*-tests and group tests according to the severity of gambling preference level, using the mean and median as the thresholds and divisions, respectively, create the pressure value below the mean sample group, the pressure value above the mean sample group, the pressure value below the median sample group, and the pressure value above the median sample group. The mean occupational fraudulent behavior tendency (*Fraud*) for each group was calculated, and the group statistics are shown in Table 3. The results indicate that the higher the level of gambling preference endured by an individual, the greater the likelihood of engaging in job-related fraudulent behavior. These findings provide preliminary support for the research hypothesis 1.

**Table 3:** Independent samples *t*-test results.

**Statistical term**	**Groups**	***N***	**Mean**	**SD**	**σ**	***F***	***t***	**df**	***P***

*Fraud*	1	284	2.750	1.563	0.093	2.650	−6.451	533	0.000
	2	251	3.668	1.729	0.109				
	3	267	2.708	1.559	0.095	2.316	−6.661	533	0.000
	4	268	3.652	1.714	0.105				

### 5.2 Correlation analysis

Table 4 presents Pearson's correlation coefficients for the main variables. The Pearson correlation coefficient between the gambling preference level (*Gamb*) and the tendency of fraud in office (*Fraud*) was 0.382, and it is significant at a 1% level. However, since the correlations only considered the relationships between the two variables without considering the influence of other factors and could not control for important control variables, the real causal relationship between stress and the respondents' reported occupational fraud needed to be further tested. From the results of the correlation analysis of the main variables, the absolute value of the correlation coefficient between most of the variables was less than 0.5, indicating that further testing is no serious problem of multicollinearity between the variables.

**Table 4:** Pearson correlation analysis of main variables.

**Variable**	***Fraud***	***Gamb***	***Ego***	***Psycap***	***Belief***	***Gender***	***Age***	***Educa***	***Works***	***Rank***	***Rev***	***Equity***	***Rat***
*Fraud*	1												
*Gamb*	0.382^***^	1											
*Ego*	0.392^***^	0.419^***^	1										
*Psycap*	−0.463^***^	−0.206^***^	−0.385^***^	1									
*Belief*	0.377^***^	0.497^***^	0.346^***^	−0.264^***^	1								
*Gender*	0.179^***^	0.148^***^	0.073^*^	−0.032	−0.029	1							
*Age*	−0.132^***^	−0.150^***^	−0.005	0.164^***^	−0.071	−0.015	1						
*Educa*	0.002	−0.003	−0.112^***^	−0.05	0.055	−0.087^**^	−0.271^***^	1					
*Works*	−0.146^***^	−0.165^***^	−0.028	0.163^***^	−0.069	0.036	0.822^***^	−0.278^***^	1				
*Rank*	0.053	−0.014	−0.01	0.038	0.058	0.178^***^	0.194^***^	−0.039	0.303^***^	1			
*Rev*	−0.039	0.04	−0.118^***^	0.118^***^	0.028	0.191^***^	0.008	0.326^***^	0.108^**^	0.301^***^	1		
*Equity*	0.022	0.016	0.009	−0.074^*^	0.078^*^	0.092^**^	−0.078^*^	0.129^***^	−0.018	−0.043	0.153^***^	1	
*Rat*	0.564^***^	0.435^***^	0.496^***^	−0.400^***^	0.372^***^	0.202^***^	−0.162^***^	−0.034	−0.149^***^	−0.019	−0.042	0.005	1

^*^p < 0.1, ^**^p < 0.05, and ^***^p < 0.01.

### 5.3 Empirical findings

#### 5.3.1 Gambling preference and occupational fraud

Following Equation (7), we examined the effects of gambling preference on office fraud, and the regression results are shown in Column (1) of Table 5. Gambling preference level (*Gamb*) and occupational fraud (*Fraud*) were significant and positive at the 1% level, with a regression coefficient of 0.173. Thus, for every standard deviation enhancement in the degree of gambling preference the probability of the respondent committing occupational fraud would increase by 15.17% (0.173 × 1.918/2.188). This indicates that the higher the degree of the actor's gambling, the greater the propensity to commit fraudulent behavior in the office, consistent with H1.

**Table 5:** Results of the test of variables related to gambling preference and occupational fraud behavior.

**Variable**	**(1)**	**(2)**	**(3)**	**(4)**	**(5)**
	***Fraud***	***Ego***	***Fraud***	***Fraud***	***Fraud***
*Gamb*	0.173^***^ (3.810)	0.244^***^ (6.687)	0.134^***^ (2.845)	0.378^***^ (3.668)	−0.056 (−0.624)
*Ego*			0.160^***^ (2.891)		
*Psycap*				−0.169^**^ (−2.108)	
Gamb ^*^Psycap				−0.039^**^ (−2.313)	
*Belief*					0.045 (0.521)
Gamb ^*^ Belief					0.033^*^ (1.938)
*Gender*	0.237 (1.386)	−0.135 (−0.983)	0.259 (1.522)	0.274^*^ (1.688)	0.342^**^ (1.999)
*Age*	0.101 (0.778)	0.019 (0.179)	0.098 (0.760)	0.149 (1.203)	0.121 (0.940)
*Educa*	0.088 (0.765)	−0.127 (−1.372)	0.109 (0.946)	0.014 (0.129)	0.063 (0.555)
*Works*	−0.140 (−1.606)	0.070 (0.999)	−0.151^*^ (−1.746)	−0.142^*^ (−1.720)	−0.157^*^ (−1.829)
*Rank*	0.394^**^ (2.485)	0.163 (1.284)	0.368^**^ (2.333)	0.340^**^ (2.266)	0.318^**^ (2.028)
*Rev*	−0.097^*^ (−1.875)	−0.056 (−1.343)	−0.088^*^ (−1.711)	−0.053 (−1.076)	−0.096^*^ (−1.882)
*Equity*	0.238 (1.217)	0.344^**^ (2.193)	0.182 (0.937)	0.061 (0.325)	0.167 (0.866)
*Rat*	0.535^***^ (11.716)	0.351^***^ (9.559)	0.479^***^ (9.708)	0.416^***^ (9.081)	0.487^***^ (10.551)
*Industry*	Yes	Yes	Yes	Yes	Yes
*_cons*	0.941 (1.461)	0.804 (1.553)	0.813 (1.267)	2.410^***^ (3.125)	1.424^**^ (2.049)
*N*	535	535	535	535	535
*R^2^*	0.397	0.380	0.407	0.463	0.420
*Adj R^2^*	0.352	0.334	0.362	0.420	0.375

*t-*statistics in parentheses,^*^p < 0.1, ^**^p < 0.05, and ^***^p < 0.01.

#### 5.3.2 The mediating role of ego depletion

Equations (8) and (9) were used to test the mediating role of ego depletion between gambling preference and occupational fraud behavior, referring to the methods of Wen and Ye (2014) to examine the possible mechanistic role of ego depletion. In the first step, the relationship between gambling preference and occupational fraudulent behavior was tested, and the regression results are shown in Table 5, Column (1), indicating that the total effect of gambling preference level on the reported implementation of occupational fraudulent behavior was positive.

In the second step, we tested the relationship between gambling preference and ego depletion as shown in Table 5, Column (2), showing that gambling preference level (*Gamb*) and ego depletion (*Ego*) were positively at the 1% level. This finding is consistent with the expectation that at higher gambling preference levels, there may be a direct negative effect. The respondent would generate more self-control to compensate for the depletion of resources in the state of pressure due to gambling, resulting in an ego depletion state.

In the third step, the effect of ego depletion (*Ego*) on occupational fraudulent behavior (*Fraud*) was examined when gambling preference level (*Gamb*) and ego depletion (*Ego*) were both entered as explanatory variables. As shown in Table 5, Column (3), *Ego* was positively correlated with *Fraud*, indicating that the higher the ego depletion, the higher the tendency to commit fraud. Following this three-step test, we concluded that ego depletion had a mediating effect on the positive correlation between the gambling preference and fraudulent behavior in the office, supporting H2.

#### 5.3.3 The moderating role of psychological capital

We examined the moderating role of *PsyCap* between gambling preference and fraudulent occupational behavior. We add two variables *Psycap* and *Gamb* × *Psycap* in Equations (7)–(10) to test H3. If the cross-multiplier term *Gamb* × *Psycap* was significant and negative, then as psychological capital was greater, the stronger the gambling preference level attenuating fraudulent behavior at work (Table 5, Column 4). The effect of the cross-multiplier *Gamb* × *Psycap* on the propensity to commit *fraud* was significant and negative, with a regression coefficient of −0.039. Thus, the moderating effect of psychological capital on the propensity for gambling preference and fraudulent behavior was supported. *PsyCap* acted as a moderating variable that attenuated the positive relationship between gambling preference level and occupational fraud behavior to mitigate the actor's occupational fraud.

#### 5.3.4 The moderating role of superstitious beliefs

This section examines the moderating role of superstitious beliefs in the relationship between gambling preference and occupational fraud behavior. We add two variables, *Belief* and *Gamb* × *Belief* to Equations (7)–(11) to test hypothesis H4. If the cross-multiplier term *Gamb* × *Belief* was significant and positive, the stronger the superstitious beliefs are, the stronger the positive relationship between gambling preference levels and office fraud would be enhanced (Table 5, Column 5). The significant positive regression coefficient for the cross-multiplier term *Gamb* × *Belief* had a significant positive effect on the propensity to commit fraud (*Fraud*) with a regression coefficient of 0.033. These results confirm that superstitious beliefs moderated the relationship between the propensity for gambling preference and fraudulent behavior in office. Thus, superstitious beliefs can strengthen the positive relationship between gambling preference level and official fraud behavior and increase the actor's occupational fraud tendencies.

### 5.4 Robustness testing

#### 5.4.1 Metrics for substitution variables

Using the mean remeasurement indicators for each item (Table 6, Column 1), gambling preference level (*Gamb_m*) and the propensity to commit office fraud (*Fraud_m*) were significantly positive at the 1% level, consistent with previous research. Examining the mediating role of ego depletion in the relationship between gambling preference and occupational fraud behavior (Table 6, Columns 2 and 3), consistent with our hypotheses. By examining the moderating role of psychological capital in the relationship between gambling preference and office fraud behavior (Table 6, Column 4), the findings are consistent with those of previous studies. By examining the moderating role of superstitious beliefs between gambling preference and occupational fraud (Table 6, Column 5), consistent with our previous findings.

**Table 6:** Results of the inferential tests showing the variables associated with the mean measure of gambling preference and the occupational fraud behavior.

**Variable**	**(1)**	**(2)**	**(3)**	**(4)**	**(5)**
	***Fraud_m***	***Ego_m***	***Fraud_m***	***Fraud_m***	***Fraud_m***
*Gamb_m*	0.180^***^ (3.968)	0.251^***^ (6.505)	0.140^***^ (3.000)	0.389^***^ (3.830)	−0.050 (−0.573)
*Ego_m*			0.156^***^ (2.998)		
*Psycap_m*				−0.159^**^ (−1.977)	
Ecopre_m ^*^Psycap_m				−0.049^**^ (−2.389)	
*Belief_m*					0.029 (0.330)
Gamb_m ^*^ Belief_m					0.042^**^ (2.017)
*Controls*	Yes	Yes	Yes	Yes	Yes
*Industry*	Yes	Yes	Yes	Yes	Yes
*_cons*	0.615 (1.207)	0.582 (1.339)	0.524 (1.035)	1.764^***^ (2.879)	1.042^*^ (1.892)
*N*	535	535	535	535	535
*R^2^*	0.389	0.378	0.400	0.457	0.412
*Adj R^2^*	0.344	0.331	0.354	0.415	0.366

*t-*statistics in parentheses, ^*^p < 0.1, ^**^p < 0.05, and ^***^p < 0.01.

#### 5.4.2 Exclusion of other industries

In the industrial statistical characteristics of this study, the “other industries” category includes 58 samples, accounting for 10.8%. Since it's not possible to specify the industries involved in the “other” category, and to prevent respondents from non-seriously scrolling down and selecting “other industries” which could degrade the overall quality of the questionnaire responses, the 58 samples that chose “other industries” were excluded from the 535 total samples. Hypothesis testing was then conducted using the remaining 477 samples. According to the test results in Table 7, the research conclusions remain consistent with the previous findings.

**Table 7:** Test results of variables related to gambling preference and occupational fraud excluding industry interference.

**Variable**	**(1)**	**(2)**	**(3)**	**(4)**	**(5)**
	***Fraud***	***Ego***	***Fraud***	***Fraud***	***Fraud***
*Gamb*	0.175^***^ (3.603)	0.208^***^ (5.481)	0.132^***^ (2.674)	0.411^***^ (3.430)	−0.057 (−0.582)
*Ego*			0.203^***^ (3.374)		
*Psycap*				−0.156^*^ (−1.697)	
Gamb^*^Psycap				−0.044^**^ (−2.270)	
*Belief*					0.070 (0.767)
Gamb^*^Belief					0.032^*^ (1.742)
*Controls*	Yes	Yes	Yes	Yes	Yes
*Industry*	Yes	Yes	Yes	Yes	Yes
*_cons*	0.931 (1.367)	0.807 (1.510)	0.767 (1.137)	2.365^***^ (2.785)	1.275^*^ (1.740)
*N*	477	477	477	477	477
*R^2^*	0.389	0.379	0.405	0.458	0.417
*Adj R^2^*	0.339	0.329	0.354	0.411	0.367

*t-*statistics in parentheses, ^*^p < 0.1, ^**^p < 0.05, and ^***^p < 0.01.

## 6 Further analysis: moderated mediated effects test

**Figure 2:**
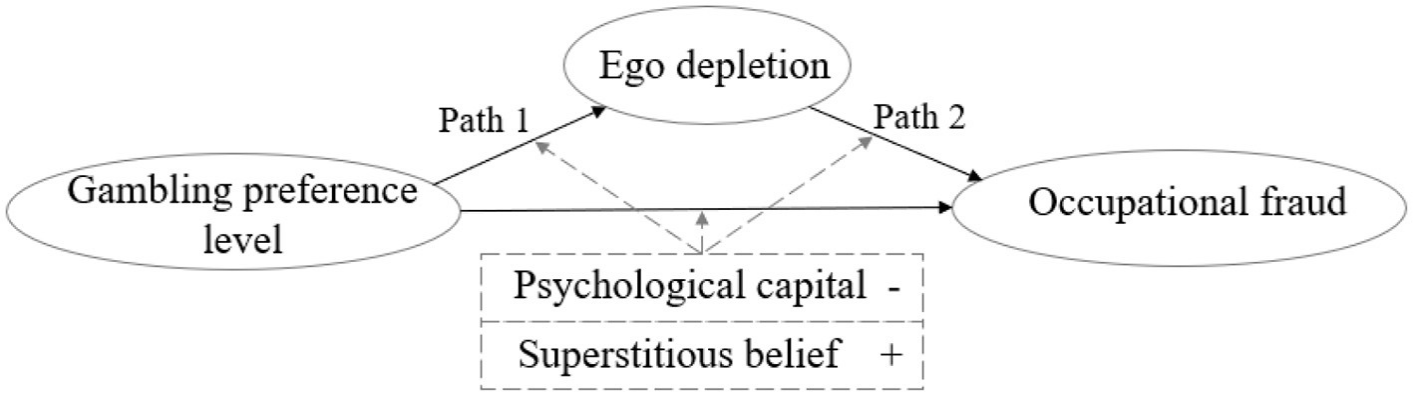
Path diagram of the moderating effect of gambling preference level mediating occupational fraud behavior.

Previous analyses in our study tested the moderating effects of psychological capital and superstitious beliefs on gambling preference and occupational fraud but did not reflect which mediating paths the moderation took occurred. The next analyses examined the mediating effects of moderation, which were tested according to the two paths of Model 1 and Model 2 as shown in Figure 2.

Table 8A shows the results of testing psychological capital as a moderating variable, and the coefficient of the *Gamb* × *Psycap* cross-multiplier term in Path 1 was −0.037 and was significantly negative at the 5% level. Thus, psychological capital attenuated positive effect of gambling preference on ego depletion. The *Ego* × *Psycap* variable in Path 2 was not significant, indicating that occupational fraud behavior resulting from ego depletion is difficult to effectively regulate through psychological capital. Therefore, Path 1, with its moderating and mediating effect, holds true. Table 8B shows the test of superstitious beliefs as a moderating variable, in which the coefficient of *Gamb* × *Belief* cross-multiplier term in Path 1 was 0.370, which was significantly positive at the 1% level; thus, superstitious beliefs can attenuate the positive effect of gambling preference on ego depletion. The *Ego* × *Belief* variable in Path 2 was not significant, indicating that it is difficult to effectively regulate the occupational fraud behavior caused by ego depletion, resulting in occupational fraud being difficult to regulate through superstitious beliefs. Thus, it can be observed that psychological capital and superstitious beliefs not only modulate the relationship between gambling preference level and occupational fraud through direct effects, but also regulate the positive relationship between gambling preference level and occupational fraud behavior through the indirect effects of Path 1, thereby validating the moderated mediation pathway.

**Table 8:** Results of mediation effect tests with moderation.

**Panel A: The moderator variable is** ***Psycap***						
**Variable**	**(1)**	**(2)**	**(3)**	**(5)**	**(6)**	**(7)**
	**Path 1 (** ***Ego*** **)**			**Path 2 (** ***Fraud*** **)**		
	**Coeff**	**SE**	**t**	**Coeff**	**SE**	**t**
*Gamb*	0.522^***^	0.085	6.167	0.539^***^	0.127	4.232
*Ego*				0.153	0.131	1.168
*Psycap*	−0.137^**^	0.068	−2.010	−0.244^***^	0.090	−2.718
Gamb ^*^ Psycap	−0.037 ^**^	0.014	−2.599	−0.046^**^	0.021	−2.214
Ego^*^ Psycap				0.004	0.021	0.208
*R^2^*	0.278			0.326		
*F*	68.165^***^			51.181^***^		
**Panel B: The moderator variable is** ***Belief***						
**Variable**	**(1)**	**(2)**	**(3)**	**(5)**	**(6)**	**(7)**
	**Model 1 (** ***Ego*** **)**			**Model 2 (** ***Fraud*** **)**		
	**Coeff**	**SE**	**t**	**Coeff**	**SE**	**t**
*Gamb*	0.177^***^	0.053	3.327	0.056	0.102	0.554
*Ego*				0.209^**^	0.106	1.974
*Belief*	0.273^***^	0.041	6.737	0.016	0.095	0.169
Gamb ^*^ Belief	0.370 ^***^	0.045	8.153	0.032	0.021	1.512
Ego^*^ Belief				0.021	0.022	0.922
*R^2^*	0.017			0.251		
*F*	11.716^***^			35.428^***^		

^*^p < 0.1, ^**^p < 0.05, and ^***^p < 0.01.

Further, this study used the Process plug-in in SPSS to test for moderated mediated effects, and after the Bootstrap method and after 1,000 samples, the results of the moderated mediated effects test are shown in Table 9. When psychological capital is low, the indirect effect of gambling preference level on occupational fraud behavior through ego depletion was 0.065 (95% CI [0.011–0.141]), and since the interval does not contain 0, the mediating effect was significant. When psychological capital was high, the indirect effect of gambling preference level on occupational fraudulent behavior through ego depletion was 0.045 (95% CI [0.012–0.095]), and the interval did not contain 0, indicating a significant mediating effect. When superstitious beliefs were low, the indirect effect of gambling preference level on occupational fraudulent behavior through ego depletion was 0.044 (95% CI [0.007–0.100]), and the interval did not contain 0, indicating a significant mediating effect. When superstitious beliefs were high, the indirect effect of gambling preference level on occupational fraudulent behavior through self-attrition was 0.123 (95% CI [0.064–0.198]), and the interval did not contain 0, indicating a significant mediating effect. Thus, the mediating effect of gambling preference level on occupational fraudulent behavior through ego depletion was significant at both high and low levels of the moderating effects of psychological capital and superstitious beliefs.

**Table 9:** Moderated mediation effect test for Bootstrap sampling.

**Type of effect**	**Moderator variable**	**Effect**	**SE**	**Bootstrap lower 95% CI**	**Bootstrap upper 95% CI**
Indirect effects of psychological capital	*Psycap-1SD*	0.065	0.033	0.011	0.141
	*Psycap*	0.056	0.020	0.021	0.100
	*Psycap+1SD*	0.045	0.021	0.012	0.095
Indirect effects of superstitious beliefs	*Belief-1SD*	0.044	0.023	0.007	0.100
	*Belief*	0.080	0.021	0.040	0.123
	*Belief+1SD*	0.123	0.036	0.064	0.198

## 7 Research conclusions and discussions

### 7.1 Research conclusion

This study refined the pressure element in the fraud triangle theory following the perspective of occupational fraud motivation. We focused on Chinese corporate actor data as gambling preference offers valuable insights for conducting an in-depth analysis of fraud motivation. We empirically examined the effect of gambling preference on occupational fraud behavior by adopting the survey research method. Our study had several key findings:

First, the influence of gambling preference level on occupational fraudulent behavior showed that the stronger the degree of gambling preference level, the higher the probability of the actor's fraudulent behavior. This might be because the irrational cognition may lead to an illusion of control and attempts to compensate for losses, thereby increasing the tendency to engage in fraudulent behavior. This might be explained by the transfer of gambling-related irrational beliefs, such as the illusion of control, into professional settings. Individuals with strong gambling preferences may overestimate their ability to manipulate outcomes in work contexts, thereby increasing the likelihood of engaging in unethical or risky behavior.

Second, the mediating role of ego depletion. This study incorporates the state of ego depletion into the explanatory framework of how stress influences occupational fraud behavior. The findings reveal that individuals with higher gambling preferences experience significantly heightened ego depletion, which further undermines their self-control and moral restraint. substantially increases the likelihood of engaging in occupational fraud. Ego depletion serves as a significant mediator between gambling preferences and occupational fraud behavior, uncovering the underlying psychological mechanisms through which gambling preferences impact fraudulent behavior. By emphasizing ego depletion as a key mechanism, this study provides an integrated understanding of how gambling-induced stress compromises moral judgment and self-control, linking personal traits with occupational misconduct.

Third, the moderating role of psychological capital and superstitious beliefs, we conducted a heterogeneity study of gambling preference on occupational fraudulent behavior. We found that the stronger the psychological capital, the weaker the positive relationship between gambling preference level and occupational fraud behavior (i.e., psychological capital can act as an inhibitory mechanism to fraud behavior). The higher the superstitious beliefs, the stronger the relationship between the gambling preference and occupational fraud. In other words, superstitious beliefs can intensify the gambling preference level and the positive relationship between fraud, increasing the possibility of occupational fraud. These findings highlight the dual role of psychological capital and superstitious beliefs as moderating factors, offering practical insights for tailoring interventions to reduce occupational fraud risk.

Fourth, we tested moderated mediation effects and found that the mediating effect of gambling preference on occupational fraud through ego depletion was significant, with significant moderating effects of psychological capital and superstitious beliefs. Path analysis revealed that psychological capital and superstitious beliefs moderated the mediating effect of gambling preference on ego depletion. Specifically, psychological capital mitigated the impact of gambling preference levels on ego depletion, while superstitious beliefs amplified this effect.

### 7.2 Research insights

This study explores the mechanisms through which gambling preferences influence occupational fraud and proposes actionable recommendations for corporate governance and HR management:

First, prioritize employees' mental health management. Research demonstrates that irrational cognition, driven by gambling preferences, increases fraud tendencies through ego depletion. To address this, companies should not only support employees' mental wellbeing through methods such as psychological counseling and health education but also focus on creating a positive and healthy work environment. A supportive work culture and stress-reducing workplace policies can help mitigate the impact of gambling-related stressors, reducing the likelihood of fraudulent tendencies. Furthermore, organizations should provide targeted resources for employees who exhibit gambling tendencies, focusing on improving decision-making skills to counteract the negative effects of irrational beliefs.

Second, enhance employees' psychological capital. Strengthening psychological capital effectively mitigates the relationship between gambling preferences and fraudulent behavior. Organizations can achieve this through targeted training programs, counseling, and team-building activities, enabling employees to better cope with stress and reducing the likelihood of fraudulent actions. Investing in psychological capital not only fosters resilience but also equips employees with tools to counteract the psychological vulnerabilities that lead to ego depletion and unethical behavior.

Third, promote rational beliefs and counteract superstitious tendencies. Superstitious beliefs amplify the influence of gambling preferences on occupational fraud. Companies should foster a culture of scientific rationality through cultural events and the dissemination of scientific concepts to guide employees toward rational decision-making. Educational initiatives aimed at debunking superstitious beliefs can help employees develop a critical perspective, reducing their susceptibility to cognitive distortions that escalate fraud risks.

Finally, establish a comprehensive behavior monitoring and intervention system. Holistic behavior management systems are critical, particularly for employees with high gambling preferences. Early identification of fraud risks through behavior analysis and employee interviews can enable timely preventive measures. Proactive monitoring and intervention systems can detect early warning signs of ego depletion and irrational behaviors, allowing organizations to intervene before these escalate into occupational fraud.

### 7.3 Research limitations and future directions

While this study provides valuable insights into the impact of gambling preferences on occupational fraud and its mechanisms, certain areas could benefit from further exploration. First, the study focuses on gambling preferences within the general employee population without distinguishing between problem gamblers and non-gamblers. This approach, while offering a broad perspective, may not fully capture the nuanced effects of factors such as gambling frequency and intensity. For example, individuals with high gambling intensity or problem gambling behaviors might demonstrate stronger tendencies toward risky or unethical actions compared to non-gamblers. Consequently, the absence of this distinction could result in a bias, potentially underestimating or overestimating the relationship between gambling preferences and occupational fraud. Second, the research examines individuals' overall occupational fraud tendency but does not differentiate between specific types of fraud, such as asset misappropriation, financial statement fraud, or corruption, which could provide more detailed insights into the mechanisms underlying the relationship between gambling preferences and fraud. Third, the study's reliance on an online survey method, while efficient and widespread, may introduce potential sample selection bias, as participation could be limited to individuals with internet access and willingness to complete the survey, which could skew the sample toward certain demographic groups. The study lacks random sampling or explicit strategies to ensure representativeness across industries and regions, which limits the generalizability of the findings. While quality checks were performed to maintain data integrity, the absence of randomization and representativeness strategies may result in unintentional biases in the dataset. Moreover, the research is conducted within the Chinese cultural context, and its findings have yet to be explored in different labor cultures or regulatory environments. Other potentially influential variables, such as job level, work stress, and employment type, were not included in the current research framework, which could enrich the understanding of occupational fraud. Additionally, the cross-sectional design and reliance on self-reported data may limit the robustness of the findings. Finally, the study does not include a direct evaluation of risk decision-making as a variable, which may independently influence the relationship between gambling preferences and occupational fraud. This aspect could slightly limit the understanding of how broader risk-taking tendencies shape fraudulent behaviors.

To address these limitations, future studies should incorporate screening mechanisms to differentiate between problem gamblers and non-gamblers, allowing for a more granular analysis of how these subgroups influence outcomes. Researchers could also explore distinctions between specific types of occupational fraud, such as asset misappropriation, financial statement fraud, or corruption, to provide more detailed insights into the underlying mechanisms. Adopting random sampling techniques or combining online surveys with other data collection methods could mitigate potential sample selection bias and improve the representativeness of the findings. Cross-cultural comparisons could validate the results and enhance their generalizability beyond the Chinese cultural context. Additionally, expanding the scope of research to include broader variables, such as job level, work stress, and employment type, as well as employing longitudinal designs and experimental methods, will further deepen the theoretical understanding and practical relevance of the findings. Finally, Future research should explore the combined effects of gambling preferences and risk decision-making on occupational fraud, incorporating risk decision-making into the research framework would provide a more comprehensive understanding of the mechanisms underlying occupational fraud.

## Data Availability

The original contributions presented in the study are included in the article/supplementary material, further inquiries can be directed to the corresponding author.

## Footnotes

^1^In this study, situational post-test items were set up to test whether the subjects understood the meaning of the questions and stayed focused during the answering process.
